# Supplementary material for: The challenges of transgender and nonbinary graduate students in chemistry: A qualitative study on trans identity, science culture, and institutional support using reflexive thematic analysis
Source: PLoS One. 2025 Apr 4;20(4):e0320493. doi: 10.1371/journal.pone.0320493 (PMC11970692; doi:10.1371/journal.pone.0320493)
Supplement: S1 Appendix — (DOCX) [file pone.0320493.s001.docx]

# S1. Appendix. Interview Questions.

Starting questions that were used to prompt conversation during the group interviews included the following:

- Please describe your gender identity, pronouns, and any other identities that are important for you.
- How did you begin choosing which universities to apply for graduate school? How did you evaluate those departments?
- Do you have any recollections from the application that stood out regarding your trans identity? (Items that stood out as supportive, as red flags, as confusing, etc.)
- Were there any questions in the application that were unclear or unanswerable?
- Did any of the programs you applied to require a diversity statement as part of the application packet? How did you respond?
- How were you addressed in follow up emails from the department after submitting your application?
- If you attended recruitment visits to campuses, how were they handled?
- How did meetings with faculty members/potential advisors go?
- What factors did you use to make your decisions when accepting or declining an offer of admission?
- What would the entry into graduate school have looked like in an ideal situation for you?
- Were support systems made clear to you during the recruitment process?
- How did you choose whether to come out as trans in the department?
- Is there anything that has not been addressed that you wish to share?
