# Supplementary material for: The challenges of transgender and nonbinary graduate students in chemistry: A qualitative study on trans identity, science culture, and institutional support using reflexive thematic analysis
Source: PLoS One. 2025 Apr 4;20(4):e0320493. doi: 10.1371/journal.pone.0320493 (PMC11970692; doi:10.1371/journal.pone.0320493)
Supplement: S2 Appendix — (DOCX) [file pone.0320493.s002.docx]

# S2. Appendix. Exchange Transcript.

To illustrate how the participants and facilitators interacted to co-construct knowledge and foster empowerment, we include here a brief example exchange from Group Interview #2.

Eris: A significant amount of programs that cater to underrepresented minorities don't consider gender identity or gender minorities to be underrepresented minorities, unless you're a cis woman. So, a lot of programs that are catering towards underrepresented minorities don't include trans people into it. That's also an indication of the federal government's idea of underrepresented minorities and how much public schools can deviate from that, but that's neither here nor there.

Michelle: I know I definitely applied for some women in science type of fellowships, and I had this internal fight with myself the whole time about like, I want this fellowship. I'm doing feminist work in science. But it's not really for me. But also, they don't understand why it's not really for me. I went back and forth with myself so many times on those.

Eris: I'm woman-adjacent enough for that. That's what I, that's what I tell myself.

Nat: Okay, back to the trans imposter syndrome again!

Eris: Don't internalize the failures of the system to harm yourself. Don’t take responsibility for that. That’s what I tell myself. It doesn’t always work.
