# Supplementary material for: The challenges of transgender and nonbinary graduate students in chemistry: A qualitative study on trans identity, science culture, and institutional support using reflexive thematic analysis
Source: PLoS One. 2025 Apr 4;20(4):e0320493. doi: 10.1371/journal.pone.0320493 (PMC11970692; doi:10.1371/journal.pone.0320493)
Supplement: S3 Appendix — (DOCX) [file pone.0320493.s003.docx]

# S3. Appendix. Exit Survey.

1. What is your chosen pseudonym? This should be the same personal pseudonym you used during the group interview.
   1. Free text response.
2. What pronouns do you use?
   1. Free text response
3. Please describe your gender identity.
   1. Free text response
4. What is the enrollment size of the institution where you are currently in your doctoral program? (Note: enrollment size typically refers to the total number of students at both the undergraduate and graduate levels.)
   1. Less than 5,000 students
   2. 5,001 - 15,000 students
   3. 15,001 - 30,000 students
   4. More than 30,000 students
   5. I have not chosen a graduate program yet
5. Reflecting on the interview, is there any information you would like to share with the researchers that was not addressed?
   1. Free text response
