# Supplementary material for: The challenges of transgender and nonbinary graduate students in chemistry: A qualitative study on trans identity, science culture, and institutional support using reflexive thematic analysis
Source: PLoS One. 2025 Apr 4;20(4):e0320493. doi: 10.1371/journal.pone.0320493 (PMC11970692; doi:10.1371/journal.pone.0320493)
Supplement: S4 Appendix — (DOCX) [file pone.0320493.s004.docx]

# S4. Appendix. Codebook.

| **Category** | **Primary Code** | **Secondary Code** | **Meaning** |
| --- | --- | --- | --- |
| In Vivo | Word of mouth | N/A | When participants collected informal information about a graduate program through conversations with peers, mentors, etc. |
| In Vivo | Hedge Bets | N/A | Calculated risks and tradeoffs in choosing a graduate program. |
| In Vivo | Women in STEM | N/A | Pressure for transmasculine people to misgender themselves for 'diversity purposes.' |
| In Vivo | TERF Women in STEM | N/A | Transfeminine people who identify with 'women in STEM' and their experiences in those spaces. |
| In Vivo | What STEM won't provide | N/A | Low expectations of chemistry departments to support trans individuals. |
| In Vivo | I'm not going to get beat up here | N/A | Uncertainty about psychological and bodily safety at a university. |
| In Vivo | Outed | N/A | Pressure to disclose trans identities or involuntary disclosure by others. |
| In Vivo | The only one | N/A | Concern about being the only trans person in a department. |
| In Vivo | Respect | N/A | Feeling respected in trans identity by the institution, department, faculty, peers, etc. |
| In Vivo | Self-advocacy | N/A | Having to advocate for autonomy, gender-affirming resources, or other recognition. |
| In Vivo | Performative | N/A | DEI actions that claim inclusivity without addressing transphobic conditions. |
| Policy | State | Bathroom Laws | Legislation restricting access to public restrooms for trans people. |
| Policy | Geography | N/A | Students' choices based on perceptions of trans-friendliness of a region. |
| Policy | Institutional | Healthcare | Health insurance and medical resources supporting trans individuals. |
| Policy | Institutional | Equal Opportunity Policy | Policies aligned with anti-discrimination laws, Title IX, etc. |
| Policy | Departmental | Support Staff | Personnel assisting students with paperwork, transitions, etc. |
| Policy | Departmental | Providing Pronouns | Culture of sharing pronouns in introductions, emails, etc. |
| Policy | Organizational | LGBTQ+ Organizations | University LGBTQ+ offices or student organizations. |
| Policy | Organizational | Other Student Organizations | Non-LGBTQ+ organizations like 'Women in STEM'. |
| Versus | Positive | N/A | Experiences perceived positively regarding trans identity. |
| Versus | Negative | N/A | Experiences perceived negatively regarding trans identity. |
| Versus | Egregious Disrespect | N/A | Extreme transphobia beyond typical negative experiences. |
| Versus | Assigned to You | Name | Use of a deadname or misgendering vs. affirmed name. |
| Versus | Lived by You | Pronouns | Whether pronouns used align with lived experience. |
| Versus | Autonomy | N/A | Whether students have autonomy in gender expression. |
| Versus | Subjugation | N/A | Experiences where autonomy was restricted. |
| Versus | Trans-Inclusive | N/A | Policies, behaviors, or places that genuinely affirm trans identities. |
| Recruitment | Emails | N/A | Email correspondence with departments. |
| Recruitment | Visit | N/A | Campus visits during graduate school recruitment. |
| Recruitment | IT System | N/A | Entry of names and gender markers in institutional systems. |
| Recruitment | Name Tags | N/A | Name tags given during campus visits. |
| Recruitment | Meetings | N/A | Meetings with potential advisors or faculty. |
| Recruitment | Accommodations | N/A | Gendered facilities like hotel rooms and bathrooms. |
| Recruitment | Q&A | N/A | Participants' questions to prospective departments and their responses. |
| Recruitment | Application | N/A | Questions asked in graduate school applications. |
| Recruitment | Gender Neutral Bathrooms | N/A | Availability and accessibility of gender-inclusive restrooms. |
