# Supplementary material for: The challenges of transgender and nonbinary graduate students in chemistry: A qualitative study on trans identity, science culture, and institutional support using reflexive thematic analysis
Source: PLoS One. 2025 Apr 4;20(4):e0320493. doi: 10.1371/journal.pone.0320493 (PMC11970692; doi:10.1371/journal.pone.0320493)
Supplement: S5 Appendix — (DOCX) [file pone.0320493.s005.docx]

# S5. Appendix. “Hedged Bets” Theme

### Summary

Herein, we share the specific information that participants used to “hedge bets” when weighing graduate programs against one another. These supporting data give insight into the participants’ process when considering graduate programs. We share these data so that departments can examine what information is being offered to prospective students. However, we caution against hyperfocusing on these specific informational items and losing sight of the big picture concerning how difficult it is for trans students to make decisions about their futures.

**S5.1 Table. Summary of specific concerns participants raised related to the “Hedge Bets” theme.**

| **Topic** | **Subtopic** | **Number of Participants** |
| --- | --- | --- |
| **Geographic location** |  |  |
|  | Discussed location broadly | 9 |
|  | Only considered a friendly region (politically liberal, diverse population), or ruled out a hostile region | 7 |
|  | Wanted to leave home or undergraduate institution area | 2 |
|  | Concerned about specific state legislation | 1 |
|  | Existing friend network in prospective city | 1 |
|  | Region supportive of mental health | 1 |
| **Institutional resources** |  |  |
|  | Healthcare policies that include gender affirming care coverage | 6 |
|  | Campus LGBTQ+ Resource Center | 6 |
|  | LGBTQ+ student organizations on campus | 2 |
|  | Chemistry department DEI work | 3 |
|  | Equal employment opportunity statements and Title IX policies that include gender identity | 2 |
|  | Gender and Women's Studies Centers | 2 |
|  | Gender inclusive campus housing | 1 |
|  | DEI mission statements from chemistry department | 1 |
| **Word of Mouth** |  |  |
|  | Perspective of current graduate students in a department | 5 |
|  | Advice from an undergraduate mentor about a department's reputation | 3 |
|  | Knew/trusted their future PI before applying to the institution | 1 |

### Geographic Location

Almost all of the participants (*N*=9) discussed geographic location as a high or highest priority factor in applying to graduate programs. Participants either explicitly named location as a factor or discussed location as their measured proxy for other priorities, such as political climate, bodily safety, or existence of local trans communities.

Many of the participants felt resigned to the fact that there would be limited or non-existent trans community inside prospective chemistry departments, leading them to prioritize “friendly” geographic areas where they felt they could find trans folks in the greater community. Alex, a genderfluid transfemme, mentioned that she wanted to live somewhere where they “would feel more comfortable living in the city and not focus on the department itself.” Kayden, who described their gender as nonbinary, genderfluid, and transmasculine, similarly said that they were “kind of a pessimist” about finding a trans-affirming department, so they valued the surrounding area more than the university itself. Farren, a trans man nearing the end of his doctoral program, was even more explicit about his low expectations for chemistry departments. Farren reflected that he was in a period of gender exploration when entering graduate school and he prioritized moving to one particular city above all other considerations. Understanding that he could not rely on any institution for the community connections he needed, Farren applied to only one graduate program in a city where he believed he could thrive. He also said that in retrospect, he had been “a little naive” to believe he would not need trans-informed support from within the department as well.

*I actually felt interested in moving to [city] specifically because of the trans population here, and because of just access to medical care, and just queer inclusivity in tons of different places. I was questioning gender at the time, but I didn't really have the language around transness yet. But I knew that I was queer and something was happening and I needed to be in a place that would feel safe. [...] I sort of just anticipated that I would not be friends with anybody in the department. I wasn't friends with anybody in my [undergraduate] department because there weren't any out queer people. [...] I wanted to ensure that I would have a community that would be able to support me through a PhD without having any dependence on the department itself. [...] I think it was a little naive thinking that I can just get away with being in an inclusive city, even if the university itself was trash.* -Farren

Other participants considered regions of the United States, rather than individual cities, as acceptable or unacceptable options for future residence. A majority of the participants (*N*=7) either said that they would only consider living in a comfortable region (e.g. politically liberal, diverse), which they considered to be a minimum indicator of safety as a trans person, or alternatively ruled out regions that they perceived to be inhospitable or dangerous (e.g. Texas, the US Southeast). In some cases, a lack of information about a specific place forced the participants to make judgment calls based on approximations from general cultural trends. Kayden said that they “mostly applied to coastal schools.” For universities located in a region with “a reputation of being not friendly,” they prioritized institutions inside cities where there was a greater likelihood of finding other trans people. In this scenario, Kayden relied on their assumptions that larger cities tend to be more diverse and more accepting to guide their geographic screening process.

Two participants (*N*=2) described wanting to leave the area in which they were raised or pursued their undergraduate degrees because they had personally faced transphobia in that area. Indigo, a trans woman who grew up in the rural US South and had experienced transphobic barriers, said that location was her highest priority when considering graduate programs. For Indigo, her previous experiences of oppression (especially in relation to healthcare autonomy) were associated with location. She believed that by moving to another place, she would have access to the trans affirming support she was denied earlier in her life. Indigo said that she “somewhat naively” applied to universities in areas “where trans people seemed to be very accepted” based on her impressions of regional trans support or oppression.

*I had come out a year before and I had not pursued any medical transition options yet. I had looked into it, but being from the rural south, I ran into many roadblocks in trying to get that. So I had not yet started. So one of the big things on my mind was how easily it would be to medically transition. And so I applied to schools, probably somewhat naively, based upon where trans people seemed to be very accepted. So I applied to some schools in [US west coast states]. I had some other schools in mind that I decided not to apply to because they were geographically located also in the south or the southeast. So, I had a school in Texas that I was really interested in and I did not apply because I did not want to be in Texas.* -Indigo

When the research team asked Indigo for more information about how she approximated which areas appeared to be accepting, she shared that she did not investigate specific chemistry departments online. Instead, she relied on information about the “more liberal overarchingly” reputations of an area and hoped the regional climate would transfer down to the departmental level itself. Indigo was nearing completion of her PhD at the time of the interview and she expressed in hindsight that this method may not have been the best way to evaluate whether a graduate program would be a supportive environment as a trans student, saying “that was not a smart idea for myself.” Although Indigo and Farren both described their assessments about geographic location as “naive” in retrospect, their reasonings were subtly different. Farren assumed that no matter how unsupportive a department or university was for trans students, living in a city with a critical mass of trans people would provide all the community support he needed. Indigo assumed that if a university was located in a trans accepting area, that the departmental politics would align with those of the region where it was located. Looking back on the application period from a more experienced vantage point, both participants saw flaws in their reasoning at the time.

Eris, a nonbinary transfemme, was currently in the process of interviewing with several graduate programs and had not yet committed to an institution at the time of the interview. In terms of location, they described how choosing institutions in which to apply was a balancing game between estimating the external, local queer community and internal, institutional queer community. Eris wanted to ensure there would be queer support networks for her, which she assumed would exist in large cities but may not exist in smaller college towns. Examining Eris’s process of sensemaking was unique because she was the sole participant who was “hedging bets” in real time, rather than reflecting on her process afterwards. Eris weighed their impressions based on trends about queer life in cities or small towns in the following way:

*One other big thing that I'm considering is the location. [...] In the middle of a major metropolitan area, I'm much less concerned about finding a queer environment at the school because there is a major metropolitan area right next door that I could escape to. If it's a university that is located in a small college town of 10,000 people, I'm much more concerned about the school having a queer community, and having those queer support networks there in the university rather than outside the university, if that makes sense.* -Eris

One participant (*N*=1) pointed to specific US state legislation as a major cause of distress during the graduate school application process. Jack, a transmasculine person who is a “from a northern place, now living in a southern place,” shared that he had one viable graduate program offer in a state where legislation limiting trans people’s access to public restrooms was under consideration. Jack was torn between going into this graduate program or not attending graduate school at all. He decided to pursue this graduate program after making a promise to himself that if the conditions were too oppressive after one year in this geographic location, he would leave and move on to other career pursuits, saying “if it was the worst thing in the world… I could leave, it's fine, I won't be better or worse for that year.” In his evaluation of location and personal liberties, Jack decided to take a risk living in a US state he knew to be hostile, but ultimately believed that his safety was more important than obtaining a doctoral degree.

*I kind of was waffling. "I should do it [enroll in graduate school]. I shouldn't do it. But it's going to be a year, just figure it out." At the time, the state that I was going to be moving to was proposing bathroom bills. And literally the legislation was happening the same like week or two after visitation weekend. And so it was just kind of that awkward time, and then it died in committee a week before I was supposed to start there. So it was just kind of in general an internally tumultuous time. That [question of restroom legislation] wasn't going to be something that anyone would be able to answer except for the state government. And so that was just kind of looming in the back of my head. Is this actually going to affect me?* -Jack

Some participants (*N*=2) described geographic location considerations that were not explicitly connected to their trans identities. For Nat, a nonbinary transfemme, choosing a location that would support her mental health was of utmost importance. She shared that she applied to campuses in numerous cities, but “I could not envision myself doing a PhD in New York City. That is an incredibly stressful environment to live in for five years.” Theo, who described hirself as genderfluid and genderqueer, said that sie did not yet identity as trans when applying to graduate school. However, sie still considered the location of personal support networks at hir prospective university. Theo applied to a specific department, sharing that “I'd already spent a summer in that department as a summer student. I'd found a job there. I already had a small number of friends in the city. I already knew some professors and grad students in the department and I liked the research.”

Anna, a trans woman, chose to pursue her doctoral studies at a university where she already had a mentoring relationship with a professor who would become her graduate advisor. Prioritizing her trust with a future PI whom she already knew trumped other considerations of the greater geographic area.

*I actually knew a professor at my current university who taught me in undergrad, but she left my undergrad institution to get a professorship at my current school. So I knew her. I was very comfortable with her. And so I basically followed her here. [...] For me, it was really the personal connection that I had with my PIs. I don't know, I felt very comfortable with the idea that I would have somebody who I knew and was comfortable with being able to go to bat for me, and being able to tell the department “Hey, this is her name. Use this name and not what you see on the forms.” All that sort of stuff. Whereas, you know if I went somewhere else, I felt like it would be more on me. And I didn't quite like that.* -Anna

While Anna was the only participant who said the geographic location itself was a lower priority factor (*N*=1), her motivations were not dissimilar to those who used location as a proxy measurement for trans support networks. For Anna, the university affiliation of one specific advisor alleviated her anxiety about having no faculty allies in her future program who would support her during her graduate studies. In the same discussion, Anna also mentioned some dissatisfaction with the greater geographic area of her institution, saying “there is no queer life in the city, unfortunately.” In this way, Anna made tradeoffs and sacrifices between local community and internal departmental support. She was ultimately satisfied with her decision, saying “There were no real issues that came up with [joining] the research group at all. I was very fortunate” and “I went there for the personal aspect, and I'm very glad I did.”

### Institutional LGBTQ+ Resources

The majority of the participants (*N*=6) discussed gender affirming healthcare as a major factor in their evaluation of institutions. One participant (*N*=1) described their personal need for gender affirming care as low because they planned to complete surgical procedures before entering graduate school, but still used the inclusion of gender affirming care in graduate student health insurance plans as an indicator of university support for trans students. Not all trans people need or want gender affirming medical care: some participants did not consider medical benefits as a factor in their decisions. Finding information about what types of gender affirming healthcare would be covered by the health insurance provided to graduate students was not always straightforward. Jack said there were universities where he “scoured” websites looking for specific insurance policies to determine “what they covered and what they didn’t cover.” Jack eliminated institutions from consideration if he could not find information about health insurance “with relative ease,” saying “if you can't find that on the school website, it's probably not going to be a good school in general for anything.” In a positive example, Alex shared that the program they chose to pursue had “pretty good health care” insurance and readily provided extra support to students pursuing transition-related medical care, including a dedicated mental health team with specialization in trans issues. Generally, the participants felt it should have been easier to find the information they needed to make decisions.

An institution having a student affairs LGBTQ+ center was also a positive indicator for many students (*N*=6): three participants explicitly mentioned an LGBTQ+ campus office as a requirement for their application, one mentioned it as a strong preference, and two participants discussed using LGBTQ+ center websites to gather context about the institution. Some participants (*N*=2) said that they looked for LGBTQ+ centers at prospective institutions, but were disappointed to find that these centers were heavily focused on the undergraduate student body and often did not present information about graduate student resources. Outside of formalized campus centers, some participants (*N*=3) looked for LGBTQ+ student organizations (e.g. oSTEM). In the group discussions, the participants said they wanted to know if these resources existed so that they would both have somewhere to seek help if they needed it and to know if there was an active queer presence on campus. For example, Kayden found reassurance that there would be other trans people at prospective institutions by looking at a campus LGBTQ+ center’s presence on social media.

*I remember scrolling through like social media, especially because I was looking at schools that had LGBT centers. If the LGBT Center social media existed and had like visibly not straight-cis people I was like, "ah, ok," it's like there's a certain level of “I'm not going to get beat up here" which is nice.* -Kayden

For Kayden, the presence of other trans people in institutional social media content indicated a some level of bodily safety on campus. In contrast, several other participants expressed skepticism about whether promotional materials were genuine indicators of a trans-friendly environment or if they were simply marketing initiatives with no substance behind them.

When looking for evidence of queer and trans support within chemistry specifically, only two (*N*=2) participants noted seeing an LGBTQ+ chemistry organization at any institution, and they both noted that it was extraordinarily rare. One participant (*N*=1), cast a wider net and looked for academic scientists doing LGBTQ+ advocacy work and then tracked those professors back to their institutions. Nat inferred that if individual faculty members were doing advocacy work, then their departments were likely informed or supportive of LGBTQ+ people.

*I did a Google search and just found articles on LGBTQ+ equity in science and I looked for names and tried to find common patterns between like which people were working on that front and what universities they were at. Because I had the idea at the time that any university that makes space for that kind of work and gives it adequate breadth of effort is probably a decent place to be.* -Nat

Some participants pointed to other policies or campus resources they used as part of their “bet hedging” evaluation. Two participants (*N*=2) looked for equal employment opportunity statements and Title IX policies that explicitly included protections related to gender identity and sexual orientation. One participant (*N*=1) looked for evidence of “queer infrastructures” on campus even if they were undergraduate-focused, such as gender inclusive housing. Two participants (*N*=2) looked for wider support of gender and women’s studies where they believed they could find allies, including women’s studies departments with tenured faculty members and campus women’s resource centers. One participant (*N*=1) looked for mission statements about diversity, equity, and inclusion (DEI) on university and chemistry department websites. For Indigo, a DEI mission statement should be a requirement for consideration, but she cautioned against putting too much faith into those statements in isolation. In her assessment, the absence of DEI language indicated hostility, whereas the inclusion of DEI language was an open question that she needed more context to evaluate.

### Word of Mouth

For many of the participants, the most valuable information for evaluating which chemistry programs would be a good fit were the personal recommendations of other people, which the participants referred to as “word of mouth knowledge,” “whisper networks,” and “academic gossip.”

Several participants (*N*=3) shared that the endorsements or objections of trusted undergraduate mentors were a chief source of information when assessing potential graduate programs. For example, Jack had disclosed his trans identity to one of his letter of recommendation writers and was able to ask this mentor about the success of other LGBTQ+ students in various programs. For Jack, knowing that other LGBTQ+ students had succeeded in a program was “a benefit,” even if he “never interacted with those people.” Jack was not necessarily looking for other contacts to befriend; he found it reassuring to know that if other LGBTQ+ students were successful in a chemistry program, he could also be successful. Nat had not yet disclosed her trans identity to her undergraduate research advisor at the time of graduate applications, but similarly approached her advisor to “vouch for the general character” of potential doctoral advisors.

*I basically went to my PI at the time and said “Hey, this is the field that I'm interested in. Can you give me a list of names that you think might be good mentors or people who are big in the field?” And he came back to me with like 20 or 30 names, so I just dug through their research portfolios on their websites and saw what interested me and also sort of the general institutions where they were at. And that's how I picked which institutions to apply to. And then, sort of, after that, I did like a more personal vetting process for PIs to figure out what is going to be the most trans friendly environment and what are good lab dynamics in my field.* -Nat

While Nat and Jack relied on the positive endorsements of their mentors, Eris shared that their undergraduate mentor “forbade” them from applying to some doctoral programs. Eris was out to their mentor as trans. She reasoned in retrospect that because her undergraduate research mentor was a gay man, he must have been trying to protect her from entering into a hostile learning environment.

*My [undergraduate] PI happens to be gay. I didn't know that at the time that I chose him as my undergraduate PI. He expressively forbade me from applying to some schools because he knew that they wouldn't be a good fit. He said “I'm not sending a letter of recommendation to this school even if you do apply.” So, I'm thankful for that. Yeah, I think he saved me from some very awkward situations.* -Eris

The participants also highly valued the perspectives of current graduate students in their prospective programs when it was possible to get in contact. Looking for evidence of “trans specific support” in a department, Nat shared that she reached out to and made friends with a current graduate who was able to answer Nat’s questions about “trans social life at the university” and the feasibility of accessing “medical benefits.” Kayden similarly sought insider knowledge from current graduate students during onsite departmental visits, saying “I made an effort to be like, ‘do you know anyone in chemistry who is trans or who uses they/them pronouns here?’ and it usually took a while but I could find like one person to talk to.”

Cameron, a nonbinary student, went through the graduate recruitment process on Zoom during COVID-19 restrictions and had a much harder time getting access to current students to ask questions. Cameron took a risk and contacted a current student by email. By coincidence, this contact was also trans and was able to help Cameron with information about the climate of the department. When the research team asked Cameron what would have been helpful during the recruitment process, they wished they had been able to speak with more students at every prospective institution. In Cameron’s case, the absence of informal, in-person meetings where they could assess the climate towards trans people was a major obstacle towards selecting a program.

Eris was also participating in many recruitment events virtually. However, Eris was interviewing as an out trans person and directly asked for trans contacts with whom they could speak with privately.

*Having an abundance of queer students on these visitations is very important. And that's usually not the case. So I tried to make it a tactic to ask both the graduate students and the professors during panels whether there is a queer population in the department, specifically asking for names, if they're open about it. Because anyone can say “yeah we have queer people,” but not everyone can say “oh yeah, this is a queer person, and I just messaged them to see if I can give you their contact information.”* -Eris

Eris described herself as taking “the sledgehammer approach” to making connections with current trans students, but it’s important to understand that not all trans students are comfortable disclosing their trans identities without building trust first. It is clear from the group interviews that speaking with queer and trans people at prospective institutions is important, but if these connections are not proactively provided by a department, prospective trans students must out themselves in order to ask for assistance, which was a cause of significant tension for the participants. Jack, who was not out in his applications, summarized this dilemma. The information he could find online did not speak to the actual climate of a department, but he also did not feel safe to disclose his trans identity in asking for the information he sought.

*I wouldn't be able to tell for sure if it was a friendly campus. Like policies are great, they're going to get you so far, but it's not like you physically know someone at each of those schools or you feasibly could contact someone at each of those schools. Like "Hey, me emailing you from this email address that clearly has a different name on it than what I'm going to tell you in this email, how would this come across, uh for a trans student?" You know? It's not something that I was really going to put myself out there and do.* -Jack

For as much weight as personal endorsements held with the participants, several of them also lamented how difficult it was to rely on “word of mouth” information. Nat summarized her frustration, saying there’s no “centralized way of knowing” what the conditions in a graduate program will be like.

*I think a lot of my issues in applying to grad school could also have been addressed generally – they overlap a lot with my concerns with picking individual labs in general, right? There is no centralized way of knowing “Oh, this is a lab where people work 70 hour weeks. Oh, this is a lab with a PI who just never gets back to anyone.” It's like, all this stuff is done through gossip and academic whisper networks, and I hate that. I wish there were just some centralized way of knowing, like, “Hey, this is a person who is actively [trans] supportive and doing good research.” I wish it would have been easier to find that, and not have to like sort through data and hunt for it.* -Nat

When the research team asked participants whether prospective departments and/or professors proactively gave indicators of trans support in their communications, the only participant who experienced a meaningful show of support was Anna, who had already developed a relationship with a future PI whom she trusted to be her advocate. Some participants (*N*=3) noticed when faculty and students at prospective departments provided their pronouns in email signatures or Zoom names, which they took as a positive sign. However, we believe the participants who mentioned disclosed pronouns in email signatures were stretching to find any indicator of support in the absence of explicit information. A much more meaningful indicator of trans support would have been departmental representatives expressly providing campus resources and information related to the trans experience. In this case, the absence of evidence in the group interviews was an important observation to the research team.

### Doctoral Research Agenda

In contrast to the predominant paradigm of beginning graduate studies, future doctoral research agendas were barely discussed by the participants. When chemistry research areas were discussed, it was always in combination with other weighted factors (e.g. location, mentor suggestion) and research agenda was not considered the highest priority for any of the ten participants.

Some participants (*N*=3) started from a list of chemistry departments based on research agendas and then whittled down their options according to their other priority factors. For example, Nat began her list of potential graduate institutions by asking her undergraduate research PI, a trusted mentor, for recommendations of future doctoral advisors in a research area. This list served as a kind of first draft, which she then used as the basis for further evaluation as she gathered more information about institutional climate and research group dynamics. Other participants first assessed institutions according to their highest priorities and then considered available research agendas at viable institutions, using chemistry research as a last filter. Kayden described that they were able to use chemistry research as a sort of tiebreaker at the end of their application process because they had already “prescreened” institutions. Kayden described that they had a list of options that they estimated had the “same amount of not coolness [...] or okay-ness” with their trans identity and pronouns, allowing them to make their final decision “based on science.”

*So because I like prescreened a lot of my places, like I mostly felt comfortable using like science and if I found an advisor who I thought, you know, “their lab group is full of people who respect me and the advisor seems chill enough to respect me and my pronouns." The one place that had gotten through my screening that, you know, was really bad turned out - had no trans people, made fun of my name - I was like, "No, you're getting x'ed out I don't care that you have cool science," but yeah I mostly felt comfortable. Because most places either like had the same amount of like "not coolness with it" or like same amount of, like, "okay-ness with it" that I could choose based on science.* -Kayden

The relatively low attention given to doctoral research agenda by the participants demonstrated that for trans students, non-academic factors are often more important for decision making than academic factors. For example, Kayden eliminated one institution where they were particularly interested in the chemistry research of the department after interactions with current students and faculty, saying that they were misgendered and their lived name was mocked. For Kayden, hostility towards their nonbinary identity precluded even the most compelling research opportunities.
