# Supplementary material for: The challenges of transgender and nonbinary graduate students in chemistry: A qualitative study on trans identity, science culture, and institutional support using reflexive thematic analysis
Source: PLoS One. 2025 Apr 4;20(4):e0320493. doi: 10.1371/journal.pone.0320493 (PMC11970692; doi:10.1371/journal.pone.0320493)
Supplement: Attachment — Submitted filename: Response.pdf [file pone.0320493.s007.pdf]

NM STATE BE BOLD. Shape the Future. New Mexico State University

Chemistry & Biochemistry
New Mexico State University

Dear Editor,

I am pleased to submit the revisions for our manuscript, *The challenges of transgender and nonbinary graduate students in chemistry: a qualitative study on trans identity, science culture, and institutional support using reflexive thematic analysis*, for consideration in *PLOS ONE*. We are very thankful for the reviewer's feedback and we believe they have helped make the manuscript better. Below are our responses to their suggested changes.

Thank you for your time and consideration. We look forward to your feedback.

Sincerely,

Paulette Vincent-Ruz, Ph.D. (she/ella)
Assistant Professor in Chemistry Education Research
pvr@nmsu.edu
Dept of Chemistry and Biochemistry
New Mexico State University
P.O. Box 30001, MSC 3C
1175 North Horseshoe Dr.
Bldg 187, Room 103
Las Cruces, NM 88003-8001

Response to Reviewers and Editor

General Comments

We thank both reviewers 3 and 4 for their time and constructive feedback to the manuscript. We wanted to start by generally addressing information from both reviewers, as summarized by the editor below:

> ***Both reviewers emphasize the need for a more comprehensive and up-to-date review of the literature, particularly incorporating broader discussions on transness in academia, STEM contexts, and intersectional perspectives. One aspect would be the provision of a clearer theoretical framework, expanded discussion of systemic inequities in academic spaces, and improved integration of the results with the existing literature. Both reviewers also highlight the importance of addressing intersectionality.***

We appreciate the reviewers' desires for a more extensive review of the literature. In previous iterations of manuscript review, we attempted to slim down the background information to its bare foundations due to reviewer concerns of excessive manuscript length. We have reintroduced a clear section explaining the theoretical frameworks we used to approach this work in this version of the article. It is the opinion of the authors (including a librarian) that a literature review as exhaustive as requested is well beyond the scope of possibility for one research article. We instead present a very intentionally scoped literature review that addresses two core questions we considered as researchers during this project: *What do existing reports tell us about the experiences of trans graduate students and/or trans STEM students? What elements of disciplinary culture and institutional conditions in chemistry are necessary to understand our participants' stories*? We believe the contemplation of these conditions give the reader necessary context for situating the participants' counterstories. We do refer to larger works for more information should readers want to explore related information.

While very worthwhile research pursuits, we are resistant to overly incorporating clinical literature perspectives in this study. We understand there is a large body of clinical research about trans people and mental health, trans affirming care, health disparities, and so on. However, these issues were not directly raised by the participants. The authors who were involved in the group interviews believe it is against the spirit of what the participants discussed to force a medicalized analysis onto their words. Our participants specifically wanted to speak truth to power about the institutional roadblocks they faced in graduate school. The participants

(and the authors) are instigating an intervention by way of institutional policy and departmental culture change, not in counseling or medical care. With this perspective in mind, we trust readers to sit with the participants' words and recognize the harm experienced without us needing to frame their stories with health outcome statistics.

For example, Nat needed medical leave approval in order to pursue transition-related care:

- *In scope:* doctoral advisor control over her time, departmental bureaucracy to get leave approved, benefits and health care policies to which she has access
- *Out of scope:* the psychological benefits of trans affirming care, trans people as medical patients, marginalization by clinical workers

We have brought more focus to intersectionality throughout the text, emphasizing the Black feminist methodology employed and the compounding impacts on multiply marginalized trans students. Finally, as two of the authors were co-constructors with the participants during the study, we have found it very challenging to separate the results and discussion in this article, or to separate the author comments from the data. However, we have made a good faith effort to disambiguate the results and discussion into separate sections, with more integration of literature throughout the discussion.

We also are genuinely glad that the manuscript draft raised so many followup questions for the reviewers and we think these trains of thought are important research directions that should be pursued by others in the future.

Reviewer #3

- **Abstract:**

1. ***Line 31. Although a structured abstract could not be required, I would suggest adding the number of respondents, mean age, and gender identity as participants represent a principal focus of your study.***

We have added a reference to the number of participants in the abstract (N=10). We did not collect data on the participants' ages and therefore cannot include a mean age in the description. The participants described their gender identities in their own words, and we do not wish to impose categorical boxes to summarize them in the abstract.

- **Introduction:**

2. ***Pag. 2, line 47. I suggest considering the use of "trans*" to keep the focus on capturing a variety of gender identities and expressions for individuals whose gender identity or expression is different from their assigned sex at birth (e.g., see Tebbe et al., 2014. Revised and abbreviated forms of the Genderism and Transphobia Scale: Tools for assessing anti-trans* prejudice) although not all of them identify as trans due to the variability of nonbinary gender (e.g., Rosati et al., 2022. Non-binary clients' experiences of psychotherapy: Uncomfortable and affirmative approaches).***

We appreciate that the reviewer sought inclusive terms to encompass the identities of trans folks and believe this suggestion was a thoughtful one. The rise of the term *trans** (with the asterisk) as a Boolean truncation term gained popularity in trans internet spaces in the early 2010s and it did indeed migrate into scholarship. However, language is fluid and shifting, and the term *trans** largely carries a negative connotation in the community presently as the truncation implies transgender, trans woman, etc. but not identities like "nonbinary" or "agender" which do not contain the same root word. Some trans folks use the asterisk and we respect trans peoples' right to self describe their experiences however they choose. We have chosen to continue using simply the term *trans* defined as an umbrella term throughout the manuscript as we believe it is the best descriptor for our study. We are also confident that the ten participants are comfortable and identify with the use of "trans" as an umbrella term, as we outlined our usage of the word trans in the call for participants.

3. ***Pag. 2, line 60. I suggest adding "men" after "cisgender and heterosexual".***

We have made this change in the manuscript.

4. ***Pag. 2, line 65. I would suggest expanding the theoretical framework to include studies on STEM in a broader context:***
   - Cech, E. A., & Waidzunas, T. J. (2011). Navigating the heteronormativity of engineering: The experiences of lesbian, gay, and bisexual students. Engineering Studies, 3(1), 1–24;
   - Patridge, E. V., Barthelemy, R., & Rankin, S. R. (2014). Factors impacting the academic climate for LGBQ STEM faculty. Journal of Women and Minorities in Science and Engineering, 20(1), 75–98.

Thank you, we have integrated these references in the theoretical framework.

5. ***Pag. 2, line 72. Given the context in which this study is situated, I would suggest considering that this is not merely a matter of humanity but also an institutional duty and a fundamental right to education first and to employment later.***

We agree completely and are happy to add language indicating as such:

> *"Confronting hostile discipline cultures and empowering trans students in STEM is a fundamental matter of human dignity, right to education, and right to employment."*

6. ***Pag. 3, line 77. I would suggest delving deeper and including additional studies on disparities in academia before addressing those in doctoral programs (e.g. Russell et al., 2016). Mental health in lesbian, gay, bisexual, and transgender (LGBT) youth).***

We have added an overview of the types of disparities trans collegians face to our theoretical framework so that readers have more context. While research on trans youth is important, we think it is less relevant to the stories of graduate students because studies on trans youth are typically situated in coming of age narratives and low autonomy/control of one's life due to age. The authors see more similarities between graduate students and trans professionals in the workforce, and have added a few references about trans workers.

Here is the overview on trans undergraduates added:

> *"In the setting of higher education, a growing body of research demonstrates the harmful impacts of cisnormative collegiate settings on trans undergraduate students, including complicated daily negotiations regarding outness [1,32], bodily safety on campus [2], access to gendered spaces [33,34], hostile social climates [35], hostile STEM classrooms [36,37], mental health challenges [38], exposure to microaggressions [39], and much more. Fully describing the oppression of trans students is outside the scope of this article, but we point readers towards review articles [40–42] and longform texts [1,43] for in depth context."*

7. ***Pag. 3, line 102. It would be better to state that they do not fully capture the experiences of trans individuals.***

We have made the reviewer's suggested change.

8. ***Pag. 3, line 114. After "decisions" I suggest adding "and act as barriers to access for these individuals".***

We have explicitly called attention to the fact that there are barriers to educational access in the text.

9. ***Pag. 4, line 116. The literature already demonstrates the impact of cis-heteronormative systems in academic contexts, including in STEM fields (e.g., Hughes, B. E. (2018). Coming out in STEM: Factors affecting retention of sexual minority STEM students). Please revise and enhance the literature review and the theoretical rationale of the study, with a particular focus on situating this research within the broader academic discourse and articulating its specific contributions to the existing body of knowledge. This involves critically examining current studies, identifying gaps or underexplored areas in the field, and demonstrating how this research addresses these gaps. Emphasizing the study's originality and relevance will reinforce its value to scholars and practitioners in the field.***

We would like to respectfully point out that the article raised in the reviewer's comment is about LGBQ undergraduate students in STEM and does not address trans people or graduate students. There is a dearth of studies regarding trans students in STEM. We have emphasized throughout the theoretical framework section that the conditions trans graduate students face are unique and have not received adequate scholarly attention or real action towards improvement. Our study makes a number of tangible barriers visible so they can be addressed.

10. ***Pag. 4, line 119. Considering the intersectional approach of the paper, I would take into account not only race as an additional layer of potential discrimination but also other factors, such as disability.***

We understand and agree with the reviewer's comment regarding the intersectional experience of oppression. We, however, did not collect this data on purpose to protect the identity of our participant's. We have however, added a specific comment about this in our methods section:

"We want to acknowledge that gender identity does not exist in isolation: trans people conceptualize their genders through the lenses of race, ethnicity, and other salient identities [76–79]. Trans people who are multiply marginalized face unique barriers, especially for trans women of color who face the most violent enforcement of trans subjugation [80,81]. While we would have liked to collect other demographic information, including the participants' racial and ethnic identities, the research team felt the risk of participant exposure would be too high due to the very small population of "out" trans graduate students in the field of chemistry."

- **Methods:**

11. ***Pag. 4, line 131. I would reformulate in "the act of sharing stories serves both as a means of data collection and as…".***

We have rephrased the sentence to make it clearer and flow better in the manuscript

12. ***Pag. 4, line 137. I would rephrase this section, as the expression "chosen pseudonyms" is unclear. It's uncertain whether it refers to the participants' chosen names as trans individuals or to names explicitly chosen for the study.***

We have updated the text to explain that the participants chose pseudonyms to use during the study itself. We did not collect the participants' real, identifiable names. To emphasize, we have never believed lived names are pseudonyms.

13. ***Pag 5, line 156. It would be more fluent eliding "Importantly".***
14. ***Pag 5, line 158. I think this part could be made more fluent by omitting the statement from "Acknowledging" to "participant".***

We have rewritten this paragraph to make the language more concise and clear.

15. ***Pag 6, line 178. It would be appropriate to justify why the participant chose a pseudonym.***

We have added a brief discussion of why the participants were instructed to choose their own pseudonyms for the purposes of the study. The following text was added to the top of the "Group Interview Procedure" section:

"Prior to the group interviews, participants were instructed to choose pseudonyms for themselves to use during the study. The rationale behind requesting participants choose their own pseudonyms included the following considerations: 1) The use of pseudonyms protected the privacy of participants from one another and from the research team during the group interview; 2) We were able to avoid the dehumanizing framing posed by participant numbers, enabling participants to refer to one another with a pseudonym organically during conversations; 3) We affirmed trans people's agency, right to self-description, and personal expression by allowing choice; 4) The research team avoided projecting our assumptions about participants by choosing pseudonyms on their behalf. Participants engaged in group discussions identified only by their chosen pseudonyms."

16. ***Pag 6, line 195. It is stated that the general steps of the RTA are being outlined, yet the specific process of the research is then discussed.***

The text has been clarified to say that we carried out the steps of RTA, explained each step. We also have included more information about the group interviews, and other methodological decisions.

- **Results:**

17. ***Pag 8, line 256. I recommend italicizing all participant quotations to enhance clarity, even if they contain just a single word.***

We have made this change throughout the document and we agree that it improves clarity. A specific call out was added to the top of the results saying "Direct quotes from the participants are presented in italicized font."

18. ***Pag 9, line 295. The number of participants should be capitalized (N=3). This comment also applies to similar situations (e.g., page 11, line 353).***

Thank you, we have corrected this mistake throughout the manuscript and supporting information.

19. ***Pag 9, line 296. In this section and several subsequent points, it appears that the authors' viewpoints and comments on the research process have been included. While these are interesting, presenting them in a separate section would be more appropriate. I would also like to point out the absence of a section dedicated to the discussion,***

which seems to have hindered the connection between the results and the existing literature.

We found it very difficult to disambiguate the results and discussion because two of the authors were involved as co-creators in the group discussions. While we think it better reflects the Sista Circles methodology to keep author interpretation alongside the data, we have tried our best to separate results and discussion sections.

20. ***Pag 12, line 405.I would suggest deepening this result by adding more literature in support:***
    - Lorusso et al., (2024). Navigating the gap: Unveiling the hidden minority stressors faced by trans and nonbinary clients in gender-affirming pathways. International Journal of Transgender Health, 1–21.

We added this reference to the section where one participant pointed out that most cisgender people do not understand how complicated and time-consuming transition related care is. Thank you for the suggestion.

21. ***Pag 14, line 479. It would be helpful to link this result to the existing literature, particularly regarding social support and transgender individuals.***

We have now created separate discussion sections for each theme. This allowed us to deepen each result and, in the discussion, more closely connect each theme to previously published work.

22. ***Pag 14, line 502. It would be helpful to deepen this result by referencing various sources in support, particularly regarding the psychological consequences of the described phenomenon (e.g. Pollitt, A. M., Ioverno, S., Russell, S. T., Li, G., Grossman, A. H. (2021). Predictors and mental health benefits of chosen name use among transgender youth. Youth & Society, 53(2), 320–341).***

We have now created separate discussion sections for each theme. This allowed us to deepen each result and, in the discussion, more closely connect each theme to previously published work.

23. ***Pag 16, line 548. It would be highly beneficial to undertake a more comprehensive exploration of the existing literature on the impact of institutionalized discrimination,***

power dynamics, and microaggressions within institutional contexts. This deeper analysis should integrate diverse and robust scholarly sources to substantiate the discussion and provide a nuanced understanding of these phenomena. Specifically, the review should examine how systemic biases and entrenched power hierarchies manifest in organizational structures, policies, and practices, perpetuating inequities. Furthermore, it is crucial to explore the pervasive role of microaggressions—subtle, often unintentional discriminatory behaviors—that cumulatively affect the psychological well-being, sense of belonging, and overall success of marginalized individuals within these settings.

Fully analyzing all the dimensions mentioned here is too large of a discussion for one research article, but we agree that the manuscript needed more. We have integrated more exploration and literature perspectives into the Discussion sections as they relate to each theme. We stayed focused on the participants' specific encounters with marginalization to draw attention to the tangible impacts of systems. We are glad that our report brought complex questions up for the reviewer and we hope it will continue to spark conversations for future research.

We have now created separate discussion sections for each theme. This allowed us to deepen each result and, in the discussion, more closely connect each theme to previously published work.

24. ***Pag 16, line 584- It would be helpful to deepen this result by referencing recognizing the chosen name (e.g., Russell et al.,2018). Chosen name use is linked to reduced depressive symptoms, suicidal ideation, and suicidal behavior among transgender youth.***

We understand that from a clinical perspective and mental health this is super important. Yes parents, caregivers, friends, teachers etc need to use chosen names and correct pronouns. This study was conducted in community, the researchers except one belong to the community. This is not parents validating a kids decisions. THis is us being in community with each other

25. ***Extracts do not accompany some parts of the results. I would suggest connecting the results with relevant extracts better. A good practice would include an extract or a reference whenever a new thematic aspect is introduced.***

We have improved the organization of the manuscript. We have also applied this suggestion in the results section in each thematic aspect.

26. ***Including a table illustrating the articulation of themes would be highly beneficial, as it would help readers better understand the thematic analysis you have conducted.***

We agree with the reviewer's suggestion and have added a table illustrating the themes at the beginning of the results section.

- **Conclusions**

27. ***It would be interesting to include the perspective of a systemic implementation of policies within academic contexts. One example could be introducing training programs for staff, such as Safe Zone. For further insight on the topic, see:***
- Evans, N. J. (2002). The impact of an LGBT safe zone project on campus climate.Journal of College Student Development, 43(4), 522–539.
- Finkel, M. J., Storaasli, R. D., Bandele, A., & Schaefer, V. (2003). Diversity training in graduate school: An exploratory evaluation of the safe zone project. Professional Psychology: Research and Practice, 34(5), 555.

We appreciate these suggestions. However, this manuscript is very specifically focused on how the structural culture of graduate school in Chemistry. While the work suggested is very important is unclear how these undergrad focused interventions could apply to our case.

Reviewer #4

1. The authors present a Sista Circles Methodology that is both conceptually exciting and suitable to the purpose and analytic intent of the study: to account for racialized gender diverse students' experiences, or, white gender diverse students' experiences, but through a framework of intersectional and trans of colour critique. Some further reflection and discussion on the relevance and significance of how black feminist methodology informs and/or serves specifically trans subjects would help. In other words, how does employing a black feminist methodology serve the trans specific focus or purpose of the study? I am wondering whether Johnson's chapter on what constitutes a trans-feminist methodology might be helpful alongside some further discussion of how Jourian and Nicolazzo's reflections on the "liberatory potential of collaborative methodological practices" : https://www.emerald.com/insight/content/doi/10.1108/s1529-212620150000020003/full/html

We thank the reviewer for pushing us to add this discussion in the manuscript so we can fully honor both the contributions of Black women to methodological practice and also to center the discussion about where current trans scholars are also pushing the field to honor trans participant voices as co-constructors of knowledge. The following excerpt is now at the top of the methods section:

> *"A central goal in our study design was to provide a space where trans chemists felt like they were active co-constructors of knowledge and research seeking the liberation of trans people. This ethos is consistent with what other trans scholars have done to continue to push research in the field as something "done for the community" rather than "done to them" [29,66]. Trans scholars have pushed for the development of transfeminist methodologies and trans epistemologies, both of which prioritize practices that lead to liberation, the center trans people's unique experiences, and affirm the notion that trans people are not broken and rather just as Nicolazzo grapples a trans epistemology where trans people "are all that exists, all that there is" [67].*
>
> *With this motivation, we found inspiration in Sista Circles methodology for the basis of our study. Sista Circles Methodology (SCM) is a Black feminist technique that fosters the collective building of counterstories by centering participant empowerment [68–71]. In SCM, participants actively engage as co-constructors in the research process and all research facilitators must be members of the community, mitigating power dynamics and challenging researcher-subject extractive dynamics [72,73]. It is crucial to emphasize that SCM shouldn't be misconstrued as merely a different name for a focus group. A central aspect of Sista Circles is a focus on participant empowerment in community, which is achieved through providing space outside the white gaze and encouraging the ways that Black women "share experiences, knowledge, wisdom, and power" to strengthen one another [72]. This intention fundamentally changes the dynamic from just an extractive data collection process to one where participants are actively working together in deconstructing oppressive experiences [74]. Furthermore, one of the central pillars of Sista Circles is the centering of Black women's unique forms of communication: Sista Circles are places where ways of expression, including the blended use of Black English vernacular, mainstream American vernacular, and nonverbal communication, are not policed or judged [72]. As non-Black researchers, the conscious choice of employing a Black feminist methodology as the basis for our study is a nuanced one. We recognize the uniqueness of SCM to serve Black women's liberation and we honor their contribution to liberatory praxis by being mindful of how we applied it to this study.*

Reflecting on SCM as a methodology allowed us to provide participants with a space to collectively build stories as a means of both healing and creating paths towards trans liberation regarding how institutions must change to allow them to thrive. In this space, participants could freely use trans community language without having to explain its meaning to cis spectators, including the insider slang and modes of expression (e.g. sarcasm) that naturally arise when trans people are together. The rationale of co-construction guided our use of a semi-structured interview protocol, encompassing starting questions vital to our research objectives while allowing participants to lead discussions towards the topics they found most important to interrogate as a group."

2. We thought it might be helpful to offer some further reflection on the elision of the students' racial identities from the analysis that is provided while still ensuring an ethical commitment to respecting the need for confidentiality. Perhaps some further discussion and macro level reflection on navigating interlocking systems of gendered and racialized power relations could be provided based on the researchers' own knowledge and insights without having to refer specifically to individual students. For example, in lines 118-119, the authors articulate that they expect the challenges experienced by trans graduate chemistry students to be "compounded by other systems of hegemonic power, especially when racialized." Can some further reflection on the intersectional experiences of racialized trans students be provided without necessarily compromising anonymity?

We understand and agree with the reviewer's comment regarding the intersectional experience of oppression. We, however, did not collect this data on purpose to protect the identity of our participant's. We have however, added a specific comment about this in our methods section:

"We want to acknowledge that gender identity does not exist in isolation: trans people conceptualize their genders through the lenses of race, ethnicity, and other salient identities [76–79]. Trans people who are multiply marginalized face unique barriers, especially for trans women of color who face the most violent enforcement of trans subjugation [80,81]. While we would have liked to collect other demographic information, including the participants' racial and ethnic identities, the research team felt the risk of participant exposure would be too high due to the very small population of "out" trans graduate students in the field of chemistry."

> ***3. The authors are clearly grounding their study and analysis in an engagement with trans-informed scholarship such as that of Nicolazzo and Jourien, for example. They also refer to and demonstrate a deep understanding of institutionalized cisnormativity. Also the authors specifically mention that they adopt "a theoretically driven approach to analysis". In light of this theoretical emphasis we recommend a more explicit articulation of the theory up front which would entail some acknowledgement of the trans informed frameworks that Nicolazzo articulates and which clearly informs the critique of cisnormativity that the authors elaborate: https://journals.sagepub.com/doi/10.1177/0042085917697203 Radi's paper on trans epistemologies might also be helpful in this respect: https://read.dukeupress.edu/tsq/article-abstract/6/1/43/137397/On-Trans-EpistemologyCritiques-Contributions-and?redirectedFrom=fulltext. The more explicit theoretical grounding of the analysis is buried in references in numbered endnotes, and so it might be helpful for the authors to bring the scholar and some of the theory into the sentences that have these footnotes. The authors do this once when they name Nicolazzo's theory, but it could be done more often. Specifically, one opportunity is in naming Ahmed's work in informing their understanding in note 76-77; the reference is technically there, but it could be brought into the sentence.***

We agree completely and have included a more comprehensive theoretical framework in this version of the manuscript, as well as more discussion of theory throughout the methodology and discussion sections. Specifically related to the foundational trans theoretical frameworks, we added this section to preface the study:

> *"We ground the present work in the call by Jourian and Nicolazzo to bring our communities to the research table and develop knowledge with trans people, rather than on or for trans people, in a collective movement towards trans liberation [29]. We aimed to work in collaboration with trans students in chemistry to collectively build counterstories [27]. The goal was to surface evidence of barriers that specifically affect trans students so institutions and chemistry departments can address them. We reject the characterization of trans people as broken or abnormal, instead turning the focus of our interrogation on the systems that enforce cisnormativity and produce material harm. Our work instigates an intervention not at the personal level (e.g. mentorship, counseling), but at the structural level through challenging harmful institutional policies and cultures. Our research team brings together expertise in chemistry education research, trans community organizing, and lived experience with the subject matter: combining theory with an analysis of material conditions allowed us to stay focused on how cisnormative systems, as Stryker describes, are "never mere abstractions; they are systems of power that operate on actual bodies, capable of producing pain and pleasure, health and*

sickness, punishment and reward, life and death" (Stryker 2006). We center and amplify the experiences of trans graduate students to make an urgent call for transformative change in graduate education during a time of rapidly intensifying trans antagonism.

4. While the facilitators in the research team contributed to the discussions in the interviews via the Sista Circles methodology to develop co-created knowledges, the authors state they did not include these contributions in the data or analysis. What is not clear is how this can be pragmatically achieved (i.e. how does one extract the prompt or the one side of a discussion to keep only participants' voices?), but also, how does this decision adhere to the tenets of Sista Circles methodology? In other words, given a commitment to the reflexive thematic analysis (RTA) what does this mean for the researchers and participants who are supposedly collaborating to produce knowledge through this study? Could the authors elaborate on this especially where if something is co-constructed, then it cannot be later extracted in single voices. We don't have a sense of how stories might have been influenced by others, added to, complemented, or evolved from what facilitators spoke about and how this informed the analysis of the data exactly in ways that exemplify or speak to RTA and the authors' methodological commitment to co-constructing knowledge. Some further reflection on the co-creation of this trans informed knowledge would further strengthen the article.

This is an excellent question, one that the research team discussed and tried to better represent in the manuscript. When we said we didn't include excerpts from the facilitators in "analysis," what we really meant was that we did not code our own words in the RTA process. The authors did not include excerpts of our own stories from the group discussion in the manuscript text itself because practically speaking, there is no way for us to have any kind of anonymity in a published version. Nolan and Blythe both still have connections to previous graduate departments, including that Blythe was a current graduate student in chemistry when this project started. However, engaging in the conversations with participants ourselves gave us a lot of insight about how to frame and present what unfolded. Our experiences of being in the group discussion influenced our coding process and interpretation of the transcripts. Even without excerpts of our own stories from transcripts, our voices are weaved throughout the analysis.

We also believe that what we personally shared was for the participants to hear, not necessarily an outside audience, as a method for building community. Of note, we told the participants that they don't need to define words or justify why their viewpoints are important. We told them to speak in regular intracommunity talk and we'd do the lifting to make it understandable to "outsiders," which the participants were explicitly grateful for because they were all tired of doing introductory education on trans topics. By sharing our own experiences, we demonstrated

our knowledge and built trust with the participants that we understood and would accurately represent the nuance of what they had to say. This was especially important for a handful of participants who had already been research participants in other studies or interviewed by the press because they expressed fatigue at talking to a brick wall. In the unrecorded wrap up at the end of discussions, the participants generally left feeling energized and hopeful this study would bring about change, rather than feeling exhausted or like their lives had been extracted for data.

For the most part, we offered starting prompts, the participants shared their perspectives, we shared some of our own experiences and how they were similar/different, and the act of sharing from all sides sparked deeper conversation among the participants that was often imbued with support for one another. Here was one example of one exchange, just to give the reviewer an idea of what the dynamics were like and how the discussions built a sense of community:

> *"Eris: A significant amount of programs that cater to underrepresented minorities don't consider gender identity or gender minorities to be underrepresented minorities, unless you're a cis woman. So, a lot of programs that are catering towards underrepresented minorities don't include trans people into it. That's also an indication of the federal government's idea of underrepresented minorities and how much public schools can deviate from that, but that's neither here nor there.*
>
> *Michelle: I know I definitely applied for some women in science type of fellowships, and I had this internal fight with myself the whole time about like, I want this fellowship. I'm doing feminist work in science. But it's not really for me. But also, they don't understand why it's not really for me. I went back and forth with myself so many times on those.*
>
> *Eris: I'm woman-adjacent enough for that. That's what I, that's what I tell myself.*
>
> *Nat: Okay, back to the trans imposter syndrome again!*
>
> *Eris: Don't internalize the failures of the system to harm yourself. Don't take responsibility for that. That's what I tell myself. It doesn't always work."*

The above conversation snippet was added to the Supporting Information. We have updated the main text in the "Group Interview Procedure" section as follows:

> *"To foster an environment where participants felt comfortable sharing their stories, the data collection process was solely facilitated by members of the research team who identified as trans, promoting a space of shared lived experience. The two facilitators shared personal stories with the group to make the conversation reciprocal and challenge the researcher/subject extractive dynamic, building trust with the participants*

through demonstrating our "insider" connection to the subject matter. Of note, we affirmed to participants that they did not need to define community terminology or justify why their viewpoints are important, asking them to speak plainly as they would with other trans folks in their daily lives. Generally, the facilitators offered starting prompts for discussion, the participants shared their initial perspectives, the facilitators shared some of our own related experiences, and the collective story sharing from all sides sparked deeper conversations among the participants. The participants led the group discussions towards the subjects they found the most pressing and raised questions to one another, giving them a voice in the research inquiry. Aligned with the tenets of Sista Circles, these conversations developed organically and were anchored in participant empowerment of one another [52]. A brief example of the dynamics is included in the Supporting Information. Although we did not code transcript excerpts from the research team members during the data analysis process described below, our experiences of engaging in conversation with the participants gave us a nuanced understanding of the data and enabled us to frame the present research in a way that is authentic to how the collective counterstory building unfolded."

Other minor points

5. ***In the abstract we are wondering if it might be best to use the umbrella term 'trans' throughout just for consistency's sake, that's all, and especially given the explanation that the authors provide in the actual article at the outset and also on pg.6 about the use of 'trans' as an umbrella term – e.g. in the first sentence 'transgender' is qualified by a range of other identificatory categories, while the final sentence of the abstract refers to 'trans and nonbinary individuals'.***

Thank you for pointing out the language choice in the abstract. We agree and have unified the language to *trans* as an umbrella term throughout the abstract and article. We chose to leave the terminology "transgender and nonbinary" in the article title to improve search findability for scholars seeking literature on trans topics in the future.

6. ***The second listed author, Blythe, does not identify their racialized identity as part of their Reflexivity Statement (line 221-227) while their colleagues Nolan and Vincent-Ruz. To ensure consistency across all the statements, Blythe could provide this information so that the depth of these statements match across all three authors***

The reflexivity statements reflect each author's reflection about which one of their identities was at the forefront while engaging with the data through analysis and manuscript writing. We believe reflexivity statements are not a laundry list of identities, rather the result of deep

reflection from each author about how their particular life experiences and biases influenced the research process. For this reason, even though we appreciate the reviewer's comment, we believe that uniformly building reflexivity statements so they look similar goes against the practice.
