# Supplementary material for: The challenges of transgender and nonbinary graduate students in chemistry: A qualitative study on trans identity, science culture, and institutional support using reflexive thematic analysis
Source: PLoS One. 2025 Apr 4;20(4):e0320493. doi: 10.1371/journal.pone.0320493 (PMC11970692; doi:10.1371/journal.pone.0320493)
Supplement: Attachment — Submitted filename: Plos ON Review FINAL.docx [file pone.0320493.s006.docx]

The article is well-written and makes a very significant contribution to the field. The data is rich and speaks to a commitment to trans desubjugation (Stryker, 2006) in the centring of the experiences of trans and binary students’ experiences in Chemistry. We support its publication subject to some minor revisions as follows:

1. The authors present a Sista Circles Methodology that is both conceptually exciting and suitable to the purpose and analytic intent of the study: to account for racialized gender diverse students’ experiences, or, white gender diverse students’ experiences, but through a framework of intersectional and trans of colour critique.  Some further reflection and discussion on the relevance and significance of how black feminist methodology informs and/or serves specifically trans subjects would help. In other words, how does employing a black feminist methodology serve the trans specific focus or purpose of the study? I am wondering whether Johnson’s chapter on what constitutes a trans-feminist methodology might be helpful alongside some further discussion of how Jourian and Nicolazzo’s reflections on the “liberatory potential of collaborative methodological practices” : <https://www.emerald.com/insight/content/doi/10.1108/s1529-212620150000020003/full/html>
2. We thought it might be helpful to offer some further reflection on the elision of the students’ racial identities from the analysis that is provided while still ensuring an ethical commitment to respecting the need for confidentiality. Perhaps some further discussion and macro level reflection on navigating interlocking systems of gendered and racialized power relations could be provided based on the researchers’ own knowledge and insights without having to refer specifically to individual students. For example, in lines 118-119, the authors articulate that they expect the challenges experienced by trans graduate chemistry students to be “compounded by other systems of hegemonic power, especially when racialized.” Can some further reflection on the intersectional experiences of racialized trans students be provided without necessarily compromising anonymity?
3. The authors are clearly grounding their study and analysis in an engagement with trans-informed scholarship such as that of Nicolazzo and Jourien, for example. They also refer to and demonstrate a deep understanding of institutionalized cisnormativity. Also the authors specifically mention that they adopt “a theoretically driven approach to analysis”. In light of this theoretical emphasis we recommend a more explicit articulation of the theory up front which would entail some acknowledgement of the trans informed frameworks that Nicolazzo articulates and which clearly informs the critique of cisnormativity that the authors elaborate: <https://journals.sagepub.com/doi/10.1177/0042085917697203>

Radi’s paper on trans epistemologies might also be helpful in this respect:

<https://read.dukeupress.edu/tsq/article-abstract/6/1/43/137397/On-Trans-EpistemologyCritiques-Contributions-and?redirectedFrom=fulltext>. The more explicit theoretical grounding of the analysis is buried in references in numbered endnotes, and so it might be helpful for the authors to bring the scholar and some of the theory into the sentences that have these footnotes. The authors do this once when they name Nicolazzo’s theory, but it could be done more often; specifically, one opportunity is in naming Ahmed’s work in informing their understanding in note 76-77; the reference is technically there, but it could be brought into the sentence.

4. While the facilitators in the research team contributed to the discussions in the interviews via the Sista Circles methodology to develop co-created knowledges, the authors state they did not include these contributions in the data or analysis.  What is not clear is how this can be pragmatically achieved (i.e. how does one extract the prompt or the one side of a discussion to keep only participants’ voices?), but also, how does this decision adhere to the tenets of Sista Circles methodology? In other words, given a commitment to the reflexive thematic analysis (RTA) what does this mean for the researchers and participants who are supposedly collaborating to produce knowledge through this study? Could the authors elaborate on this especially where if something is co-constructed, then it cannot be later extracted in single voices.  We don’t have a sense of how stories might have been influenced by others, added to, complemented, or evolved from what facilitators spoke about and how this informed the analysis of the data exactly in ways that exemplify or speak to RTA and the authors’ methodological commitment to co-constructing knowledge. Some further reflection on the co-creation of this trans informed knowledge would further strengthen the article.
5. **Other minor points**

- In the abstract we are wondering if it might be best to use the umbrella term ‘trans’ throughout just for consistency’s sake, that’s all, and especially given the explanation that the authors provide in the actual article at the outset and also on pg.6 about the use of ‘trans’ as an umbrella term – e.g. in the first sentence ‘transgender’ is qualified by a range of other identificatory categories, while the final sentence of the abstract refers to ‘trans and nonbinary individuals’.
- The second listed author, Blythe, does not identify their racialized identity as part of their Reflexivity Statement (line 221-227) while their colleagues Nolan and Vincent-Ruz. To ensure consistency across all the statements, **Blythe could provide this information so that the depth of these statements match across all three authors**

While we have provided quite detailed feedback, we do not consider the revisions to be extensive and but are offered in the support of helping the authors to further refine what we consider to be an excellent and much needed contribution to the field.

**Cited Reference**

Stryker, S. 2006. “(De)subjugated Knowledges: An Introduction to Transgender Studies.” In *The Transgender Studies Reader*, edited by S. Stryker and S. Whittle, 1–17. New York: Routledge.
